# Primary cultured fibroblasts derived from patients with chronic wounds: a methodology to produce human cell lines and test putative growth factor therapy such as GMCSF

**DOI:** 10.1186/1479-5876-6-75

**Published:** 2008-12-01

**Authors:** Harold Brem, Michael S Golinko, Olivera Stojadinovic, Arber Kodra, Robert F Diegelmann, Sasa Vukelic, Hyacinth Entero, Donald L Coppock, Marjana Tomic-Canic

**Affiliations:** 1 Department of Surgery, Division of Wound Healing & Regenerative Medicine, New York University School of Medicine, New York, NY USA; 2 Tissue Engineering, Regeneration, Repair Program, Laboratory of Tissue Repair, Hospital for Special Surgery of the Weill Medical College of the Cornell University. New York, NY, USA; Present Address: Department of Dermatology, Miller School of Medicine, University of Miami, Miami, USA; 3 Department of Biochemistry and Molecular Biology, Virginia Commonwealth University Medical Center. Richmond, VA, USA; 4 Ross University School of Medicine, Dominica, West Indies; 5 Coriell Cell Repositories, Coriell Institute for Medical Research, Camden, NJ, USA

## Abstract

**Background:**

Multiple physiologic impairments are responsible for chronic wounds. A cell line grown which retains its phenotype from patient wounds would provide means of testing new therapies. Clinical information on patients from whom cells were grown can provide insights into mechanisms of specific disease such as diabetes or biological processes such as aging.

The objective of this study was 1) To culture human cells derived from patients with chronic wounds and to test the effects of putative therapies, Granulocyte-Macrophage Colony Stimulating Factor (GM-CSF) on these cells. 2) To describe a methodology to create fibroblast cell lines from patients with chronic wounds.

**Methods:**

Patient biopsies were obtained from 3 distinct locations on venous ulcers. Fibroblasts derived from different wound locations were tested for their migration capacities without stimulators and in response to GM-CSF. Another portion of the patient biopsy was used to develop primary fibroblast cultures after rigorous passage and antimicrobial testing.

**Results:**

Fibroblasts from the non-healing edge had almost no migration capacity, wound base fibroblasts were intermediate, and fibroblasts derived from the healing edge had a capacity to migrate similar to healthy, normal, primary dermal fibroblasts. Non-healing edge fibroblasts did not respond to GM-CSF. Six fibroblast cell lines are currently available at the National Institute on Aging (NIA) Cell Repository.

**Conclusion:**

We conclude that primary cells from chronic ulcers can be established in culture and that they maintain their *in vivo *phenotype. These cells can be utilized for evaluating the effects of wound healing stimulators *in vitro*.

## Introduction

Chronic wounds are defined not by their duration in time, but by their multiple physiologic impairments to healing [1-3]. Etiologic factors of chronic wounds such as neuropathy in persons with diabetes [4], venous reflux [5], or compression of skin [6] are defined by more than 100 molecular and cellular impairments, such as inadequate angiogenesis [7], impaired innervation [8], impaired cellular migration [9] and abnormal keratinocyte activation and differentiation[10]. A more accurate term than "chronic wound" would be "physiologically impaired wound".

Pressure ulcers and foot ulcers in persons with diabetes are serious problems that can result in amputation, sepsis, and even death without adequate intervention. Persons with type 1 and type 2 diabetes have a 9.1% risk of developing a foot ulcer in their lifetime, [11] and the presence of an ulcer increases their risk of lower extremity amputation almost 6-fold[12]. The 5-year survival rate for patients with diabetes after major amputation is approximately 31%[13]. Venous stasis ulcers and their infectious complications have not been well quantified but in our experience result in numerous admissions across multiple medical services. Debridement has become the standard-of-care in patients with diabetes and a foot ulcer, pressure ulcers and venous ulcers, to remove necrotic and infected tissue and stimulate healing. In this study, we used debrided tissue from venous ulcers as the basis to investigate the cellular basis of impaired healing.

Various growth factors play a role in coordinating cellular processes involved in wound healing. Platelet Derived Growth Factor-BB (PDGF-BB) accelerates healing in part by stimulating epithelialization and granulation tissue formation [14]. Chronic wounds also demonstrate decreased angiogenesis at the local level [15]. Angiogenic growth factors such as Vascular Endothelial Growth Factors (VEGF) [16] (VEGF-c in mice); (VEGF-165), [17] Granulocyte Macrophage Colony Stimulating Factor (GM-CSF), [18] and Epidermal Growth Factor (EGF) [19] are known to stimulate wound healing. In order to understand how else GM-CSF might be involved in epithelialization and their non-angiogenic mechanisms of action, we studied their effect on fibroblast migration.

Establishing cultures of fibroblasts from chronic wounds for *in vitro *testing, although challenging, has been successful for venous, pressure and diabetic foot ulcers. The first studies of venous ulcers showed different morphology as well as impaired fibroblast proliferation as shown by punch biopsies from the wound edge as compared with normal dermis [20]. Subsequent studies showed wound fibroblasts grew significantly slower than control fibroblasts taken from the same patient and the level of cellular fibronectin was consistently higher in all wound-fibroblasts[21]. Fibroblasts cultured from venous ulcers have reduced collagen production response when stimulated with TGF-β [22] and reduced proliferative response with PDFG-BB [23] as compared with controls. Fibroblasts have been isolated from venous stasis ulcers for *in vitro *assay to evaluate cell cycle protein expression (p21) and modulation by basic fibroblast growth factor (bFGF) [24]. Pressure ulcers have not been as widely studied but cells grown from the wound bed exhibited slower proliferation as compared to control skin[25].

Cultured fibroblasts from wounds in patients with diabetes have been evaluated for mitogenic response with a variety of growth factors [23,26] and show a lower rate of proliferation when compared with normal skin. [27,28] Beginning with morphological studies, previous investigators have successfully performed a variety of assays on cultured cells from venous ulcers[21,23]. Other investigators have evaluated various combinations of growth factors to see which stimulate mitogenic response and found that combinations of PDGF-AB-IGFI, bFGF-PDGF-AB and EGF-PDGF-AB elicited the highest response [26]. Taken together, these studies support the notion that cells from chronic wounds can be cultured and biologically evaluated.

To date, novel therapeutic modalities are being tested in animal models, such as ob/ob, db/db, NOD (non-obese diabetic) mice and pigs. However, the specific pathogenesis that occurs in the chronic ulcer has not been successfully re-created in any of these models. Therefore, we focused on establishing primary cell cultures originating from actual patients and establishing cellular tests that can help evaluate potential therapy on target wound cells. In this report, we demonstrate that cells grown from patients' wounds exhibit specific biological properties that depend on their origin within the wound. Moreover, these cells appear to maintain a distinct phenotype in culture, suggesting that they can be used as a tool to test potential therapeutic agents.

## Methods

### Obtaining specimens of venous ulcers

After Institutional Review Board approval was obtained at all institutions, human tissues from debrided venous ulcers were used in the study. Debrided tissues from 4 patients (mean age of 53.5 ± 18.8 years (AVG ± SD) at the time of specimen collection) were obtained using standard sterile surgical techniques.

**Figure 1:**
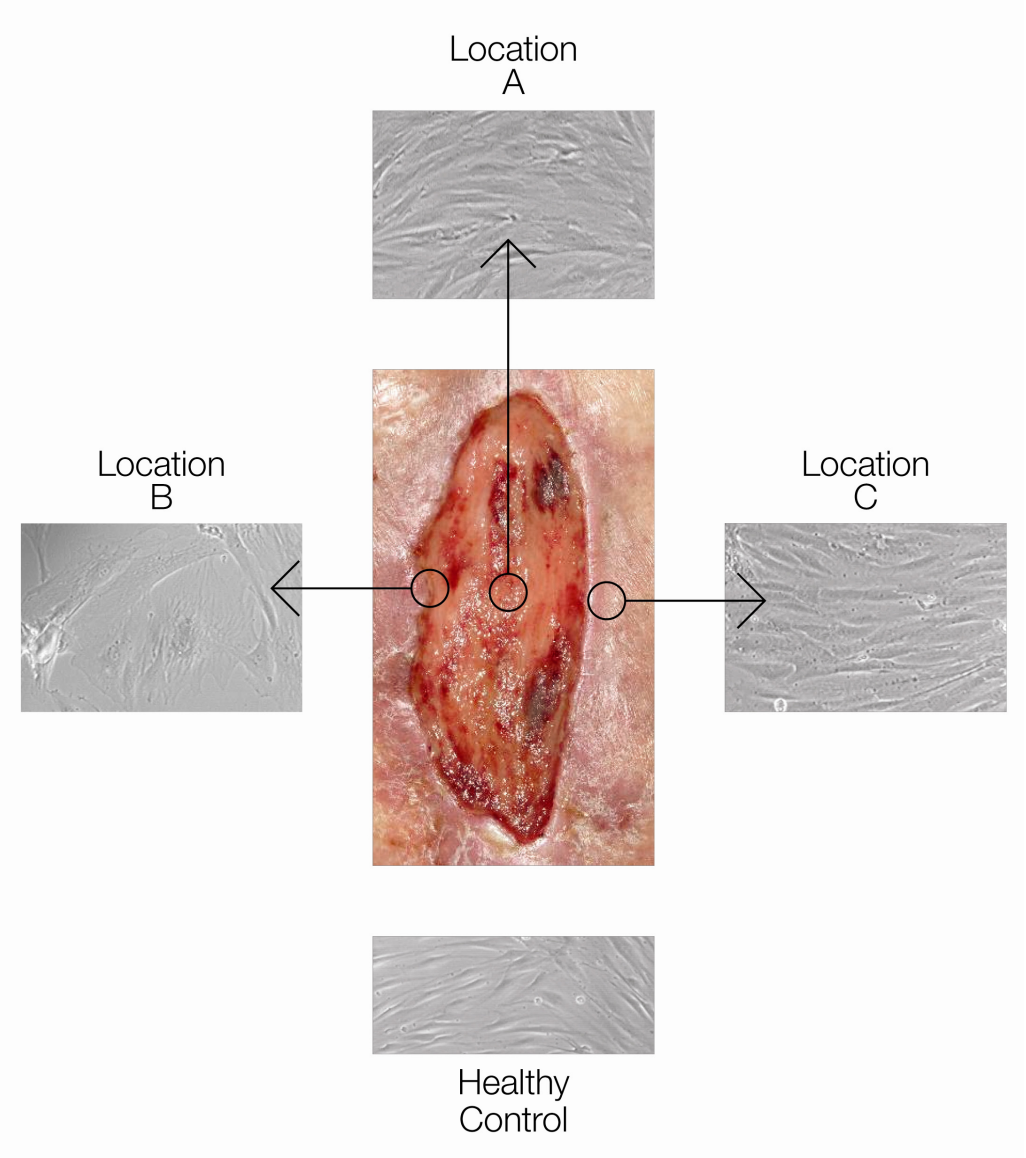
**Fibroblast deriving from different location of the wound exhibit different morphology**. The picture of the wound is shown in the center. Circles indicate origin of specific locations from which biopsies were taken. Fibroblasts deriving from each location are shown. Cells from location B exhibit different phenotype (larger in size; clumped) whereas cells from Locations C and A exhibit phenotype similar to normal healthy fibroblasts.

The area of the wound was prepared with Betadine (Purdue, Stamford, CT). Three specific areas of the wound were biopsied. A sterile #10 blade when was used to biopsy the wound base, Location A. Then Location B was identified at the boundary of the wound bed and the rim of necrotic or infected tissue to be removed. This area is often identified by a callus. After biopsy of Location B, a sharp excision was performed using to remove all the entire circumferential ring of necrotic, nonviable scar or infected tissue. Finally, a fresh blade was used to biopsy several millimeters of adjacent non-wounded tissue, (Location C, also known as the healing edge of the wound) (see Figure 1). Cells from location B are those surgically removed and cells from location C are the cells left behind after surgery. One piece of the debrided tissue was sent for routine pathology and other sections were immediately processed for cell culture. Another portion of these tissues were sent directly to the Aging Cell Repository at Coriell Institute for Medical Research (Camden, NJ). Cells derived from all four patients were subjected to tests described below.

### Cell migration assays

By using techniques previously described by us [9] and others [29] we grew fibroblasts from the wound base (location A), the non-healing edge (location B) and the healing edge (location C) and compared their migration capacities with normal primary dermal fibroblasts (obtained from mammoplasty). Cells were grown in (DMEM) (Bio Whittaker) containing 10% calf bovine serum and 2% antibiotic – antimycotic (Gibco). Twenty-four hours prior to the experiments cells were switched to basal media – Phenol Red Free (DMEM) media (Bio Whittaker) supplemented by 2% charcoal – pretreated, bovine serum as previously described [30] 1% antibiotic-antimycotic (Gibco) and 1% L-glutamine (Cambrex Bio Science). Prior to the scratch, cells were treated with 8 μg/ml Mitomycin C (ICN Biomedicals, Emeryville, CA) for 1 hour (to inhibit cell proliferation) and washed with basal media. Scratches were performed as previously described. [31] Cells were incubated in the presence or absence of 100 ng/ml GM-CSF (R&D Systems) or 25 ng/ml of EGF (Gibco) for 24 and 48 hours and re-photographed 24 hrs after the scratch. Fifteen measurements were taken for each experimental condition and expressed as a percent of distance coverage by cells moving into the scratch wound area for each time point after wounding.

### Preparation for cell culturing

Additional tissue from was sent to Coriell in 14 cc of Dulbecco's Modified Eagle Medium (DMEM), supplemented with 10% fetal calf serum (FCS), 4× Penicillin/Streptomycin, and Gentamicin in 15 cc sterile tubes. They were shipped overnight to Coriell at ambient temperature. Routine histology was performed on a portion of all biopsies.

As part of the National Institutes on Aging (NIA) Cell Repository at the Coriell Medical Institute for Medical Research (Camden, NJ) fibroblast cultures were established from the debrided tissue samples from patients with chronic wounds. Fifteen biopsies were sent to Coriell along with de-identified patients' medical history, history of diabetes, age, sex, ethnicity, status of lower extremity ischemia, and location of the biopsy.

### Fibroblasts derived from patients

Fibroblast cultures were developed according to the standard procedure of the NIA Aging Cell Repository. Once received, the biopsies were examined and, if large enough, a portion was reserved as a Specimen Quality Control sample for future use. The biopsies were finely minced with two scalpels and placed in a T25 flask in a small volume of medium. For the establishment of the culture, DMEM supplemented with 15% fetal calf serum, penicillin (100 U/ml), Streptomycin (100 μg/ml) and Gentamicin (50 μg/ml) was used. The flask was inverted and 4 ml additional medium was added. This facilitated the rapid attachment of the cells from the biopsy to the flask.

After at least 4 hours (up to overnight), the flask was returned to the upright position and the cells were cultured for 5–7 days until they were 80% confluent. Cultures were fed every 2–3 days. The fibroblasts were then subcultured by a rinse with Puck's saline with EDTA followed by incubation with Puck's/EDTA/Trypsin. An equal volume of growth medium with serum was added, cells were spun down, resuspended and plated in growth medium without antibiotics.

After an expansion in antibiotic free media, cultures were frozen in liquid N_2_. To test for viability and sterility, a vial was recovered from the freezer, passaged five times and tested for mycoplasmal, bacterial and fungal contaminants.

### Sterility testing

Each culture was tested for mycoplasma using four tests, PCR detection [32], staining using Hoechst dye, culturing for Mycoplasma in broth [33], and culturing for Mycoplasma on plates [33] Bacterial contaminants were detected using the Gram Stain. No determination of the species of bacteria was made.

### Genotyping with microsatellites assures cell line identity and culture purity

To insure the identity of each sample, all freeze recoveries and expansions of a cell line are genotyped, as well as tested for species (human or non-human, based on the presence of a specific Long Interspersed Nuclear Element (LINE)) and gender.

The development of genotyping methods provides the Coriell Cell Repositories (CCR) with the means to identify and track cell lines through all of the operations necessary to establish the cultures.

CCR has established an extensive program of genotyping based on microsatellite polymorphisms. Six highly polymorphic microsatellites have a combined matching probability of one in 33,000,000 for unrelated individuals. The characteristics of each marker are provided in Table 1.

**Table 1:** Characteristics of microsatellite markers.

**Microsatellite Marker**	**Range of Allele Sizes (bp)**	**Heterozygosity**	**pM (matching probability)**
THO-1	154–178	0.77	0.086; 1 out of 12
D5S592	166–206	0.83	0.051; 1 out of 20
D10S526	182–266	0.84	0.017; 1 out of 59
vWA31	127–167	0.81	0.062; 1 out of 16
D22S417	172–213	0.85	0.039; 1 out of 25
FES/FPS	206–234	0.67	0.165; 1 out of 6

Also available online at

The alleles of all cell lines were determined by sizing on the Applied Biosystems 3730, downloaded to the Repository database, and compared to those already recorded to assure correct identity. Gender determination was made using the amelogenin marker. Additional genotyping using Applied Biosystems AmpF/STR Identifier system using 15 microsatellite markers (including the 13 Codis markers) is used if required.

## Results

### Fibroblasts derived from biopsies of patients with venous ulcers exhibit pathogenic phenotype specific for the wound location

We found that fibroblasts chronic ulcers exhibit specific morphological changes consistent with those previously published[28]. The fibroblasts were larger in size and breadth and clumped together, whereas in the control, normal primary dermal fibroblasts were spindle-shaped (Figure 1).

**Figure 2:**
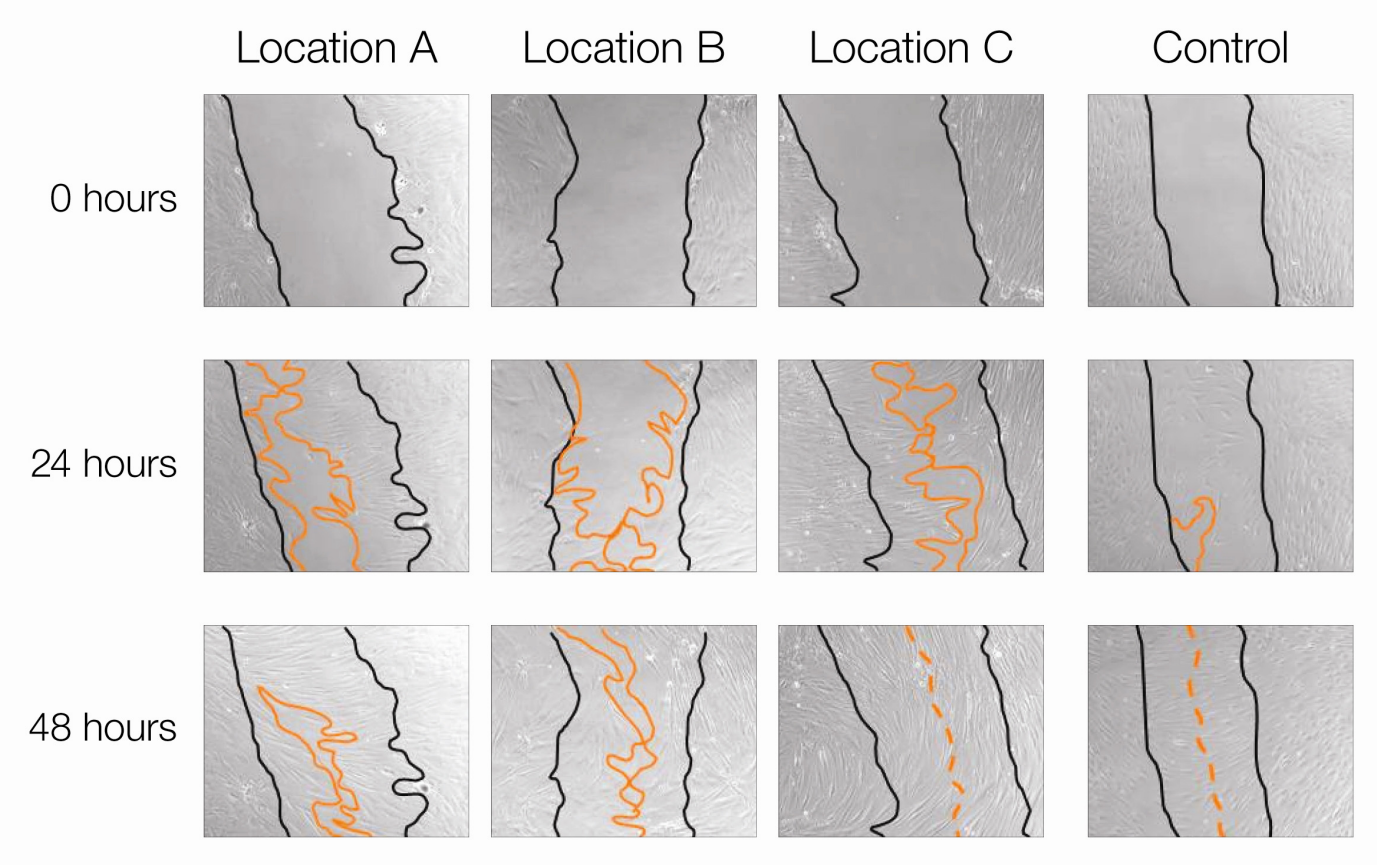
**Cells from different wound locations exhibit distinct migration capacity**. Wound scratch assay is shown. Cells from Location C migrated equally to the healthy control whereas cells from Location B have the slowest rate.

We found that fibroblasts from four venous ulcers originating from different locations in the wound migrate more slowly than control cells (Figure 2). Furthermore, we found that fibroblasts from various locations migrate differentially. Cells from healing edge (location C) migrate faster than either wound base or non-healing edge fibroblasts. Cells from the wound-base (location A) migrate faster than non-healing edge cells (location B). Thus, cells from distinct locations within the wound have distinct migration capacities reflecting their specific phenotypes.

### Human recombinant GM-CSF accelerates migration of specific fibroblasts in the wound

**Figure 3:**
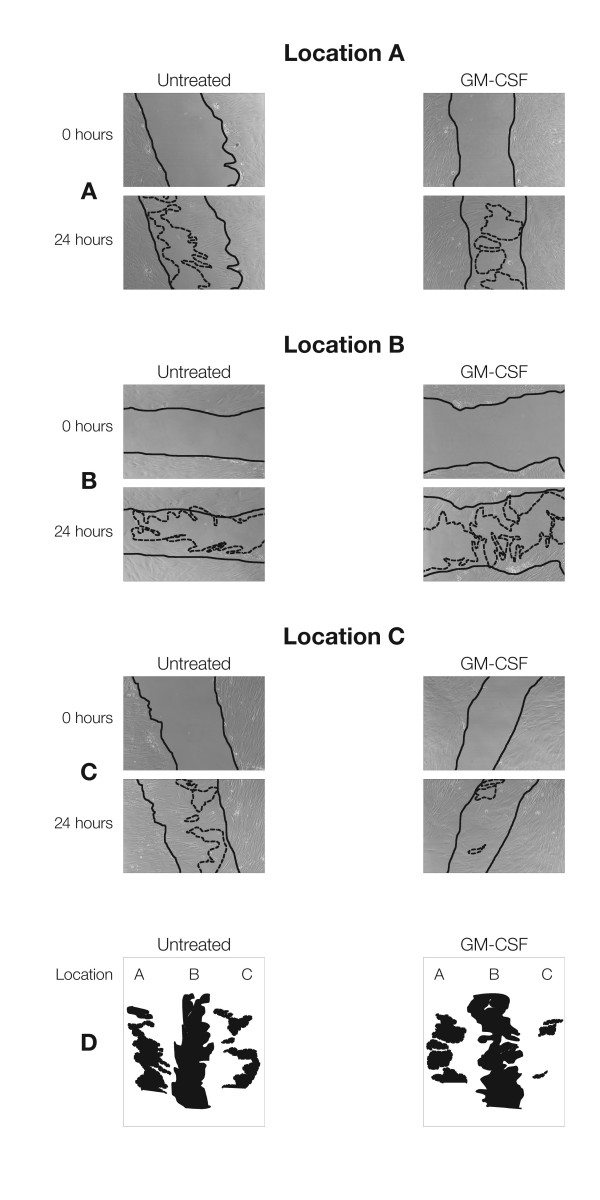
**Human Recombinant GM-CSF Accelerate Migration of Fibroblasts deriving from Location C**. Full lines indicate initial wound area; dotted lines demarcate migrating front of cells. GM – CSF treatment of fibroblasts deriving from location A (A) and location B (B). GM – CSF treatment of fibroblasts deriving from location C stimulated migration the most. (D) Surface area not covered by fibroblasts from scratch wounds are shown. GM-CSF markedly reduced wound area of fibroblasts from location C.

To determine if GM-CSF stimulate migration of these fibroblasts we used *in vitro *scratch-wound assays. Cells derived from distinct wound locations were incubated in the presence and absence of human recombinant GM-CSF. Their response to wound healing stimuli was location specific. We found that GM-CSF was the most effective in stimulating migration of fibroblasts deriving from Location C, followed by those from Location A. Fibroblasts from the non-healing edge (Location B) were not responsive (Figures 3A, B and 3C, D). EGF was used as a negative control, a growth factor to which fibroblasts do not respond in this assay [note they do respond in many other ways]. EGF did not have an effect on any of the cultures (data not shown).

### Human fibroblast cell line from chronic wounds

To establish whether the primary fibroblasts derived from chronic wound biopsies maintained their functional and structural features we grew fibroblasts from three locations in and around a chronic wound. Thirteen cultures were frozen; one sample was contaminated before freezing and one did not grow. Of these 13, 11 cultures were shown to be viable and uncontaminated. To assure viability and sterility, a vial was recovered from the freezer and passaged 5 times and then tested for mycoplasmal, bacterial and fungal contaminants. Six cultures are currently available to the research community through the NIA Cell Repository, )

## Discussion

Human fibroblast cell lines derived from patients with chronic wounds were developed and future use along with clinical data may provide information on specific aspects of disease mechanisms involving particular primary cells derived from a wound. We utilized these cell cultures to assay putative therapies for wound healing, *i.e*., gene therapies, utilizing GM-CSF as an example. We found that cells grown from specific wound locations have distinct phenotypes and diverse capacities to respond to wound healing stimuli, such as GM-CSF. Fibroblasts from the healing edges were found to be most responsive, cells from the wound base had moderate response, and cells from the non-healing edge showed minimal response. As a result of this study, 6 fibroblast cell lines, along with clinical data from patients with non-healing wounds are available to researchers performing similar assays via the NIA Aging Cell Repository at Coriell. [34]

The reduced response of non-healing edge cells is not surprising, as the cells appear to retain their phenotype *in vitro*. It is surprising, then, that GM-CSF stimulated migration of these cells. GM-CSF is known as one of the major growth factors that stimulates multiple cell types during wound healing. Studies have shown that by acting on keratinocytes GM-CSF promotes epithelialization and wound closure. In addition, GM-CSF may stimulate production of Fibronectin, Tenascin, Collagen I and alpha-smooth muscle actin [35-37]. *In vitro *studies have demonstrated that GM-CSF increases migration and proliferation of endothelial cells suggesting a role in angiogenesis[38]. GM-CSF is chemotactic for macrophages to the wound site, but such effect on fibroblasts is novel. This new finding sheds light on additional mechanisms of these growth factors in wound healing and suggests that GM-CSF has multiple functions in wound healing in addition to already established effects on angiogenesis.

Determination of the cellular response to growth factors based on their location in the wound can guide surgeons as to where to debride. Necrotic tissue impedes normal healing. Sharp debridement with a scalpel is both the most effective and readily available treatment to remove necrotic tissue and in the process removes cells that cannot respond as well to growth factors, *i.e*., cells from the non-healing edge of the wound [3]. Debridement should proceed until only the cells cultured from the post-debridement edge – those that have the ability to respond to growth factors or cellular therapy – remain. Obviously, growing primary cells from each debrided non-healing wound to guide debridement in operating room may not be practical. However, once these studies are completed and based on cellular responses one determines the location of responsive cells within non-healing wound, such knowledge would lead to determination of morphological parameters that can be used in operating room. These cells generally correspond to hyperkeratotic and parakeratotic tissue as determined by pathology results. In this fashion, a "response margin" can be established in a wound. Biopsies of tissue and their subsequent cell cultures would define this response margin and indicate further debridement. For the surgeon, findings presented here are important as they illustrate the mechanism of debridement at the cellular level and provide important evidence for incorporating this procedure in treatment protocols.

Determination of how actual human wound cells respond to growth factors may provide important information as to the potential efficacy of these potential therapies. Further, it would establish data that could be used to expand the scope of the current research and ultimately lead to a clinical trial.

The best proxies for testing on the wound are cells from the wound itself. It is evident from the literature that many different assays, such as measurement of growth factor production and response, expression of cell cycle proteins, and cell morphology, hold a piece of the puzzle as to why certain wounds do not heal. Part of the challenge is obtaining the best model to test potential therapies. The fact that fibroblasts retain their distinct phenotype in culture supports their use to test putative therapies. Although the cultured fibroblasts retain their phenotype in vitro we are currently investigating how long the cell line fibroblasts retain their phenotype through propagation.

Using the techniques described researchers can grow fibroblasts from multiple locations in the wound, the healing edge and non-healing edge to test putative therapies. Although, this study highlights cells from venous ulcers, researchers can use a similar methodology to culture cells from pressure ulcers and diabetic foot ulcers. Also, recent study has shown that fibroblasts established from the superficial dermis contains heterogeneous population of cells that has distinct morphology and proliferation kinetics [39].

The National Institute on Aging Cell Repository at Coriell is the first containing cells strains derived from chronic wounds. By using the methodology as described here, researchers can produce their own cell lines from chronic wounds in a standard fashion. These cell lines can provide clinically valuable information on cells derived from chronic ulcers.

## Competing interests

This work was supported by Grants No. K08DK0594(HB), R21DK0602214(HB) and NR08029 (MT-C), AG030673 (M.T.-C.), N01AG02101 (DC) from the National Institutes of Health and by A.D. Williams Foundation of Virginia Commonwealth University (RFD), otherwise the authors have no competing interests.

## Authors' contributions

MTC and HB conceived of the study and MTC and RD devised the experimental design for the scratch assays. HB harvested the wound tissue in the OR and HE helped in logging de-identified clinical data and delivering the specimens to MTC. MTC supervised OS and SV to carry out the culture the cells in-vitro and perform the scratch assays. A portion of the biopsies were sent to DC who led the team which created the fibroblast cell lines and made them available. AK drafted the final version of the manuscript and figure legends. MSG revised the figures, added critical content to the discussion and was responsible in revising all portions of the submitted portion of the manuscript.
